# Enhancement of Photoluminescence Properties via Polymer Infiltration in a Colloidal Photonic Glass

**DOI:** 10.3390/molecules29030654

**Published:** 2024-01-30

**Authors:** Andrea Chiappini, Davide Faccialà, Nina I. Novikova, Samim Sardar, Cosimo D’Andrea, Guido Scavia, Chiara Botta, Tersilla Virgili

**Affiliations:** 1 Istituto di Fotonica e Nanotecnologia—CNR, IFN and FBK Photonics Unit, Via alla Cascata 56/c, Povo, 38123 Trento, Italy; 2 Istituto di Fotonica e Nanotecnologia—CNR, IFN, Piazza Leonardo da Vinci 32, 20133 Milano, Italy; davide.facciala@cnr.it; 3 The Photon Factory and School of Chemical Sciences, The University of Auckland, Auckland 1142, New Zealand; nina.novikova@unimelb.edu.au; 4 Dipartimento di Fisica, Politecnico di Milano, Piazza Leonardo da Vinci 32, 20133 Milano, Italy; samim.sardar@iit.it (S.S.); cosimo.dandrea@polimi.it (C.D.); 5 SCITEC—CNR, Via A. Corti, 20133 Milano, Italy; guido.scavia@scitec.cnr.it (G.S.); chiara.botta@scitec.cnr.it (C.B.)

**Keywords:** photonic glasses, quantum yield, F8BT, photoluminescence properties, optical features

## Abstract

Photonic glasses (PGs) based on the self-assembly of monosized nanoparticles can be an effective tool for realizing disordered structures capable of tailoring light diffusion due to the establishment of Mie resonances. In particular, the wavelength position of these resonances depends mainly on the morphology (dimension) and optical properties (refractive index) of the building blocks. In this study, we report the fabrication and optical characterization of photonic glasses obtained via a self-assembling technique. Furthermore, we have demonstrated that the infiltration of these systems with a green-emitting polymer enhances the properties of the polymer, resulting in a large increase in its photoluminescence quantum yield and a 3 ps growing time of the photoluminescence time decay Finally, the development of the aforementioned system can serve as a suitable low-cost platform for the realization of lasers and fluorescence-based bio-sensors.

## 1. Introduction

Photonic crystals (PCs) and glasses (PGs) are materials with a well-known capability of affecting the propagation and reflection of electromagnetic radiation depending on the structural features, the material’s refractive index, and wavelength of light [1,2]. 

In recent years, considerable effort has been devoted to developing suitable three-dimensional photonic crystals designed for applications that require complete light confinement, such as the inhibition of spontaneous emission [3,4,5] or the development of sensors [6,7,8].

Among the different fabrication techniques, the chemical assembly approach based on the arrangement of dielectric spheres in specific ordered crystallographic structures provides a simple and cost-effective means of generating large-scale photonic crystals known as opals (or colloidal crystals) [9,10,11]. These systems are commonly employed for creating chromatic sensors [12,13], tailoring the spectroscopic features of chromophores embedded in the structures [14,15], or developing compact lasers [16,17]. 

However, due to the low refractive index contrast between the materials that constitute the colloidal crystals, these structures present a pseudo-photonic band gap. Therefore, light can be manipulated only for specific directions (generally Γ-L in the reciprocal space) [18]. This implies that the optical response of the colloidal crystal is angle-dependent, and thus, the wavelength position of the band gap can be tailored by varying the incidence angle of the light or the observation position. A complementary structure that does not suffer from this feature is the photonic glasses (PG). 

In this case, different configurations can be exploited. (a) One possible approach concerns the use of mesoscale heterogeneities in a multicomponent glass–ceramic composite. For example, Y. Yu et al. [19] demonstrated that by manipulating the mesoscale heterogeneities of the active matrix, they were able to tailor the photon emission of materials such as Li_2_O-Ta_2_O_5_-Al_2_O_3_-SiO_2_. (b) An alternative approach is based on monolayer particles assembled in a disorderly manner. By tailoring the degree of order (disorder) in the arrangement, it is possible to tune the light–matter interaction. In ordered systems, interaction with light occurs via collective modes, giving rise to diffraction phenomena, while completely disordered systems are dominated by Mie resonances of individual particles and random scattering. In this context, S. G. Romanov et al. [20] demonstrated that it is possible to tailor the disorder of a monolayer of colloids using a binary combination of “small” and “big” particles, decreasing the global order and increasing the average distance between the large scattering particles. (c) Another configuration involves the use of three-dimensional colloidal glasses based on particles assembled in a totally disordered arrangement. The advantages of these systems are associated with the possibility of having direction-independent spectral selectivity [21,22]. Moreover, since PGs are formed by monosized spheres, all the building blocks possess the same Mie resonances. This allows, in first approximation, the description of the interaction between light and the PG structure by Mie theory [23]. In this way, when light travels through the PG system, certain photons undergo stronger scattering by being trapped in a Mie resonance, reducing their transport mean free path and enhancing the time spent inside the structure. 

Recently, the features of the PGs have been exploited in various fields, such as colored covers for solar energy harvesting [24], the creation of multi-color rewritable paper [25], or the realization of photonic lignin structures with tunable and stimuli-responsive structural color [26]. 

At the same time, PG systems have been considered model systems to probe fundamental phenomena in condensed matter physics, such as light propagation [27], localization [20], and emission in active media [28]. 

In this article, we present the realization of colloidal photonic glass based on random distributions of monodisperse spheres. To study how this structure controls light diffusion in the visible range, we deposited a thin film of Poly(9,9-dioctylfluorene-alt-benzothiadiazole) (F8BT) on top of it. F8BT is widely used as a green-emitting polymer for various applications [29,30]. We compared the lifetime of the photoluminescence emitted by the F8BT with and without the photonic structure. This comparison allowed us to measure how long the light emitted by the polymer is trapped in the structure, producing a kind of cavity feedback that modifies the photoluminescence (PL) lifetime and bandwidth of the polymer and increases its PL quantum efficiency from 25% to 37%.

## 2. Results and Discussion

### 2.1. Realization and Morphological—Optical Properties

**Figure 1:**
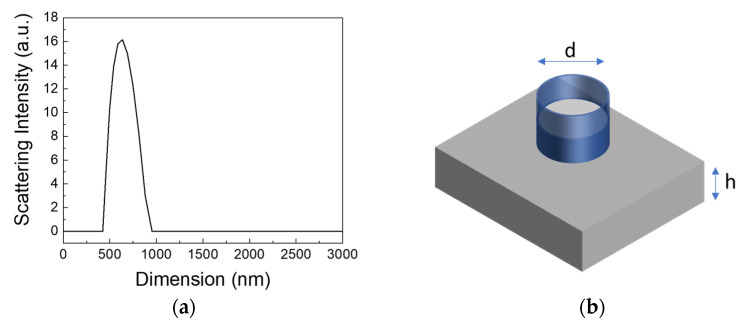
(**a**) Dynamic light scattering (DLS) spectrum showing the particle size distribution in the water solution, with a mean size value of 750 nm and a dispersity of 10%. (**b**) Schematic of the photonic glass growth method, constituted by a glass cylinder fixed with impermeable gum to a clean, hydrophilic glass substrate. The cylinder is then filled with a charged colloidal suspension previously prepared and shaken under ultrasound.

The realization of PG was achieved using monosized nanoparticles (NPs) with a dimension of 750 nm, where their size was determined through dynamic light scattering measurements (see Figure 1a). The growth of the PGs was accomplished through the electrostatic self-assembly (ESA) deposition method (sketched in Figure 1b), wherein a glass cylinder was filled with a charged suspension previously prepared, and it was left to evaporate in an oven at a constant temperature of T = 45 °C.

**Figure 2:**
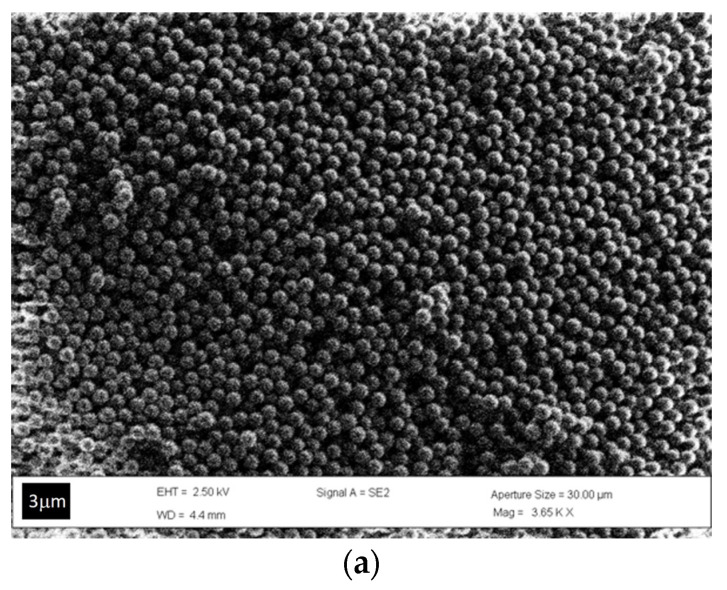

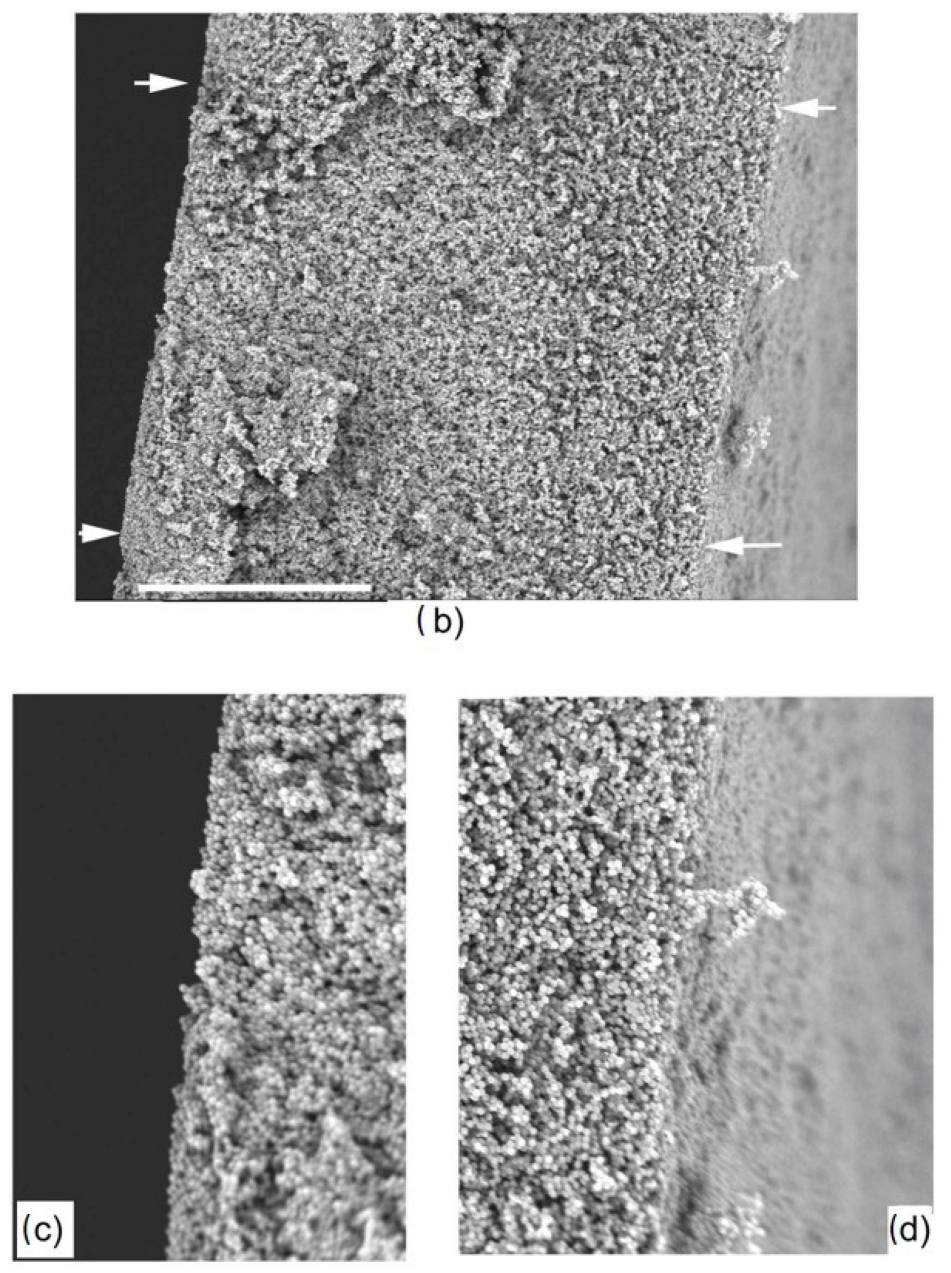
(**a**) Top-surface SEM image, where spheres of 775 nm diameter are arranged randomly by the deposition process employed; the scale bar is 3 μm. (**b**) Cross-section SEM image of a PG (scale bar: 50 microns), where the disordered distribution of the polystyrene (PS) nanoparticles involves the whole flake-like sample even at the more external levels. Arrows indicate the edges of the two surface faces. (**c**,**d**) Details of the two faces showing that PG NPs organization is preserved also on both the surfaces.

In order to confirm the random assembly of the PS NPs, scanning electron microscopy (SEM) measurements were conducted. Figure 2a shows a representative micrograph of the disordered structures. In Figure 2b, it is evident that the disordered arrangement is present throughout the entire thickness of the PG. To quantify the effective disordering, we analyzed the 2D Fourier transform patterns of the SEM images displayed in Figure 2 (see Figure S1 in Supplementary Materials).

As demonstrated by P. D. García et al. [27], the addition of electrolytes to the suspension attenuates the surface charge and enhances the number of effective collisions between spheres, increasing the formation of clusters in the suspension. This results in the creation of random closed packing, as evidenced in Figure 2. Simultaneously, it is worth mentioning that the disordered colloidal system appears entirely white, indicating that its “color” can be attributed to “random” multiple light scattering (see Figure S2 in Supplementary Materials). This feature is investigated through static transmittance spectra with an integrated sphere, measuring the total diffuse light transmission through photonic glass slabs of two different thicknesses (L1 ~100 μm and L2 ~220 μm), formed by spheres of approximately 750 nm.

**Figure 3:**
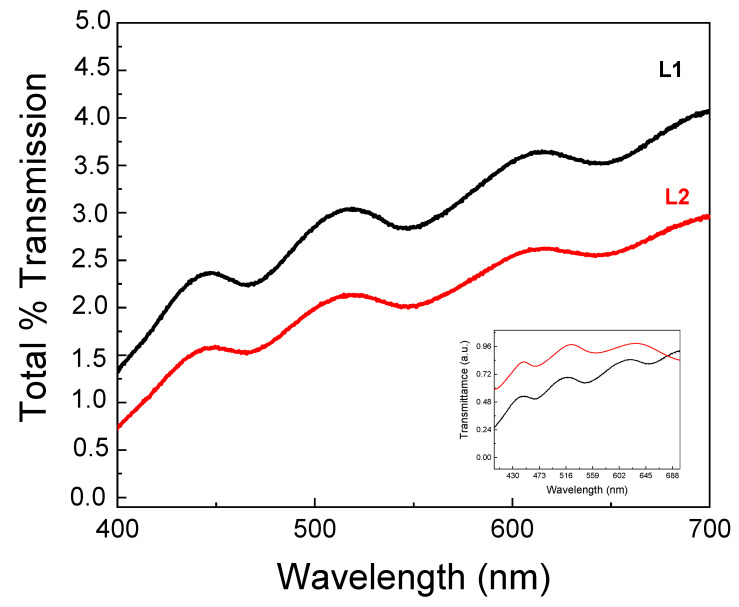
Total transmission of white light through photonic glasses as a function of the thickness of the samples L1 (black curve 100 μm) and L2 (red curve 220 μm). In the inset, the comparison between the Mie scattering response numerically obtained by a single particle model (red line) and the experimental response of L1 (black line) is shown.

Figure 3 shows the transmittance spectra of L1 and L2, where it is possible to observe the presence of oscillations in the spectra associated with the existence of modes for the electromagnetic field in the spheres (Mie resonances). The wavelength position of these resonances depends on the sphere diameter (d) and the refractive index (n) of the material.

In order to confirm this assumption, we compared the numerically obtained Mie scattering by a single particle model with the experimental optical response of the L1 photonic glass. From the spectra reported in the inset of Figure 3, an excellent agreement is observed, indicating that the diffuse transport of light is strongly affected by the Mie modes associated with the NPs that constitute the system. Finally, upon analyzing Figure 3, it is noticeable that the total light transmission decreases as the thickness of the slabs increases, in agreement with the photonic Ohm’s law. As expected, the spectral positions of these Mie modes remain unchanged. 

### 2.2. Morphological Characterization of the F8BT/PG Structure

To effectively demonstrate that this type of photonic glass can modify the spectroscopic features of chromophores, we deposited a thin film of Poly(9,9-dioctylfluorene-alt-benzothiadiazole) (F8BT) on top of the L1 sample. F8BT is a copolymer of 9,9-dioctylfluorene (F8) and benzothiadiazole (BT) units. It was initially developed as an emitter material for use in organic light-emitting diodes and has since become a model polymer [31]. In addition to its excellent light-emitting properties, F8BT also exhibits high electron mobility of 10^−3^ cm^2^ V^–1^ s^–1^ [32]. The infiltration of F8BT into the photonic glass structure was achieved by preparing a dichloromethane (DCM) solution with a concentration of 10 mg/mL. The solution was then spin-coated onto the PG structure at a rotation speed of 1500 rpm. Knowing that DCM could dissolve the PS nanoparticles, we employed a spin procedure to minimize the damage to the PG structure. The drop was placed on the substrate while it was already in rotation, aiming to increase the speed of solvent evaporation.

To understand how this procedure affects the PG structure, we conducted SEM analysis, acquiring images simultaneously with both back-scattered and secondary electron detectors in mixed mode. A fragment of the sample was detached from the glass substrate, placed vertically on a cross-section sample holder, and fixed with carbon tape. 

**Figure 4:**
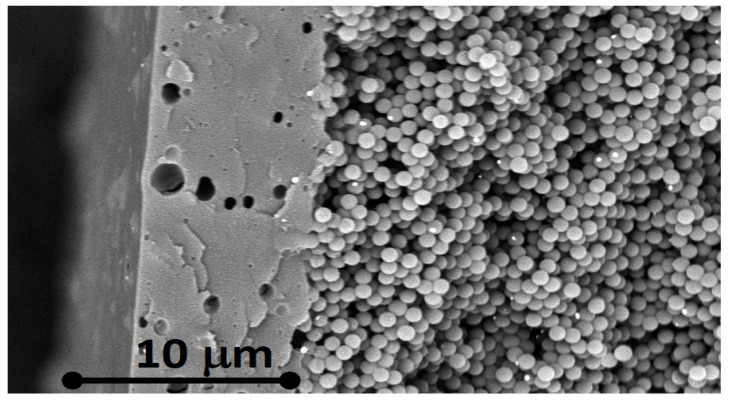
Cross-section of the F8BT/PG structure. The scale bar is 10 μm.

Figure 4 shows the SEM cross-section of the F8BT/PG structure. It is possible to observe that, after deposition of F8BT, the PG NP distribution is partially modified in the more external 5–6 microns of the PG layer, and it is substituted by a compact layer, likely due to dissolution/coalescence of the PS NPs induced by the DCM solvent of F8BT. The solution dissolves the nanoparticles, penetrating deep inside the structure, until the complete evaporation of the DCM probably leaves the F8BT polymer in contact with the remaining NPs, the deeper layer unchanged at the interface (the PG structure is 100 μm thick). In Figure 4, it is possible to see the double layer and the sharp interface between the surface compact region (5–6 μm deep) of F8BT-coalesced PS and the unchanged PS NP structure. 

### 2.3. Spectroscopical Features of F8BT 

**Figure 5:**
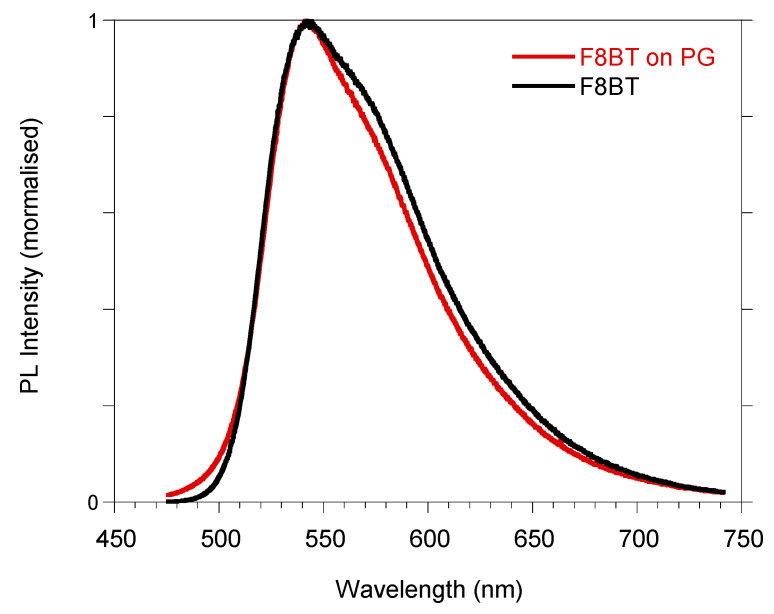
Photoluminescence normalized spectra of F8BT film spin-coated on silica (black line) and F8BT/PG (red line).

Spectroscopic characterizations were performed on our sample. Photoluminescence (PL) measurements were obtained after excitation at 405 nm using a diode laser. In Figure 5, the PL spectra of F8BT/PG (red line) and F8BT (black line) film deposited onto a silica substrate are compared. The PL spectra show the same maximum position at 543 nm; however, the emission of the polymer deposited onto the PG structure is characterized by a narrower spectral width, with reduced intensity of the long wavelength shoulder at about 570 nm. This suggests that the PG is acting on the emission features of the F8BT. In fact, the full width at half maximum (FWHM) of the PL spectrum from the polymer thin film is 340 meV, while it becomes 320 meV when the polymer is placed on the PG substrate. At the same time, it was also verified that the PG influences the emission of the F8BT by analyzing its PL decays through time-resolved time-correlated single photon counting (TCSPC) measurements (see Supplementary Materials). An increase in the lifetime of F8BT deposited on the PG system is observed compared to the one deposited on the v-SiO_2_ flat substrate. The average lifetime increases from approximately 1 ns to 1.5 ns (see Table 1). The increase in the lifetime values could be associated with a better distribution/arrangement of the F8BT chains in the PG structure, producing a modulation of the spontaneous decay rate, and/or its partial blending with the NPs dissolved during the spincoating procedure. 

This feature was confirmed by means of PL quantum yield (QY) measurements, reported in Table 1. The F8BT polymer deposited on the PG structure possesses improved PL properties, with a PL QY enhanced from 25% to 37%. 

To better understand the role of the PG structure in determining the PL variations observed for the F8BT polymer, we performed femtosecond time-resolved fluorescence measurements using a commercial fluorescence upconversion setup (Halcyone, Ultrafast Systems) on the F8BT and F8BT/PG samples with excitation at 485 nm and a temporal resolution of 200 fs. 

**Figure 6:**
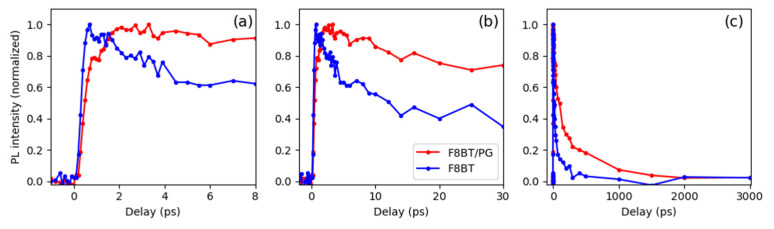
Time-dependent PL signal at 545 nm (region of integration 535 nm–555 nm) obtained from fluorescence upconversion measurements in F8BT (blue line) and F8BT/PG (red line). Different temporal windows are shown: up to 8 ps (**a**), up to 30 ps (**b**), and up to 3 ns (**c**).

Figure 6 compares the time-dependent PL intensity (integrated in a spectral region between 535 nm and 555 nm) obtained for F8BT (blue line) and F8BT/PG (red line) in different temporal windows. The first temporal window shows up to 8 ps, the second up to 30 ps, and the last up to 3 ns.

These data confirm a longer lifetime of the F8BT emission deposited on the PG with respect to a flat v-SiO_2_ surface (see Figure 6b,c). In fact, the PL decay from the F8BT/PG structure (red line) presents a longer component compared to the thin polymeric film deposited on a silica substrate (blue line). Moreover, the higher time resolution compared to the time-resolved TCSPC measurements allowed us to resolve the initial growing time of the PL intensity, which was found to be around 3 ps for the F8BT/PG sample and instantaneous (with respect to our 200 fs time resolution) for the film deposited on the silica substrate (see Figure 6a).

To understand this initial growing time, we need to consider multiple scattering of light as it travels through a PG structure [33]. Therefore, straight or ballistic propagation, which is characteristic in a homogeneous medium, cannot accurately describe its transport. The total transmission of light is given by two different contributions: the first comes from the photons that follow shorter optical paths and are transmitted at earlier times, and the second contribution comes from the photons that perform longer random walks and emerge at much longer times. This produces a time spread of the initial pulse of light, which depends on the diffusion constant. To characterize the photonic structure, we performed time-resolved transmission measurements using a streak camera with a 3 ps time resolution. We used a pump pulse at 750 nm with a 140 fs pulse duration. The result is shown in Figure S4 in the Supplementary Materials. 

The time trace of the pump pulse transmitted through the PG structure does not show any broadening or time delay with respect to the instrument response, indicating that the time spread of the initial pump pulse is shorter than the instrumental time resolution of 3 ps. This means that the initial growing time shown in Figure 6a for the F8BT/PG sample, being around 3 ps, is indeed an effect due to the light traveling in the PG structure producing a kind of “light trapping” in the structure. This interpretation, supported by the observed slight narrowing of the PL bandwidth (see Figure 5) and the large increase in the PL QY, indicates that the light emitted from the polymer infiltrated in the PG structure is amplified by a “cavity-like effect”, which induces an amplified spontaneous emission effect.

## 3. Materials and Methods

PG realization: PS spheres were synthesized by free emulsion polymerization [34] with an average diameter of 775 nm and a polydispersity of 10% measured with a dynamic light scattering (DLS) apparatus (Litesizer 100 purchases by Antoon Paar (Graz, Austria)). The z-potential of the PS spheres used in this work has a surface potential of –26 ± 1 mV, measured with the electrophoretic mobility of the spheres. Then, to the initial polymeric colloidal suspension, 10 μL of aqueous CaCl_2_ suspension with a concentration of 0.01 M were added and shaken using an ultrasound bath for 5 min to force the flocculation of the spheres. The realization of the PGs occurred through an evaporation-assisted sedimentation (EAS) deposition method using v-SiO_2_ as a substrate. Three milliliters (sample L1) or five milliliters (sample L2) of the charged colloidal suspension were allowed to evaporate on the v-SiO_2_ substrate overnight at 45 °C. 

From a morphological point of view, the disordered arrangement was verified using scanning electron microscopy (SEM) (JEOL-JSM 6300, Tokyo Japan), while the optical properties of the PGs were evaluated by transmittance measurements coupled by an integrated sphere using a double-beam Varian spectrophotometer (Carry 5000, Santa Clara CA, USA).

Film preparation. Poly[(9,9-dioctylfluorenyl-2,7-diyl)-alt-co-(1,4-benzo-{2,1′,3}-thiadiazole) (F8BT) was purchased from American Dye Source, Inc. F8BT films were obtained by spin-coating a dichloromethane solution at 1500 rpm (c = 10 mg/mL), which is flooded on the v-SiO_2_ substrate or dropped on the PG system. The morphology of the F8BT/PG structure is verified using a Phenom Pro Desktop scanning electron microscope (Thermo Fisher Scientific Inc., Eindhoven, The Netherlands) at an accelerating voltage of 5–10 kV.

Scanning electron microscopy measurement cross-section analyses. These were performed by using a Phenom Pro Desktop scanning electron microscope (Thermo Fisher Scientific Inc., Eindhoven, The Netherlands) at an accelerating voltage of 5–10 kV by acquiring the images simultaneously with both back-scattered and secondary electron detectors in mixed mode. PG and PG/F8BT flakes were detached from the substrates, placed vertically on a cross-section sample holder, and fixed on carbon tape.

Photoluminescence. PL spectra were obtained with a NanoLog composed of an iH320 spectrograph equipped with a Synapse QExtra charge-coupled device by exciting the sample with a monochromated 450 W Xe lamp. The spectra were corrected for the instrument response. PL QY values were measured with a home-made integrating sphere, following a procedure reported elsewhere (Ref. [35]), by exciting the sample with a 405 nm Thorlabs DL5146-101S laser diode with an LDC205C controller.

Time-resolved Time-Correlated Single Photon Counting (TCSPC) measurements were obtained with a PPD-850 single-photon detector module and a DeltaTime series DD-405L Delta Diode Laser. Data were analyzed using the instrument Software DAS6 6.8.0.10, employing bi-exponential best fits, with average lifetimes obtained as τ_av_ = $\frac{\sum_{n=1}^{2}\alpha_{n}\tau_{n}^{2}}{\sum_{n=1}^{2}\alpha_{n}\tau_{n}}$, as reported in the Supplementary Materials.

Fluorescence upconversion measurements were performed using a commercial setup (Halcyone, Ultrafast Systems, Sarasota, FL, USA) on the F8BT and F8BT/PG samples after excitation at 485 nm. Femtosecond pulses were generated using a Ti:Sapphire system consisting of an oscillator (Vitara, Coherent Inc., Santa Clara, CA, USA) and a regenerative chirped pulse amplifier (Legend Elite, Coherent Inc., Santa Clara, CA, USA), providing pulses of ~130 fs duration, 800 nm center wavelength, and 3 W average power at 1 kHz repetition rate. The output beam was split into excitation and gate beams. The excitation pulse at 485 nm was generated using an optical parametric generator, TOPAS-C (Light Conversion Ltd., Vilnius, Lithuania). A neutral density filter was used to attenuate the intensity of the pump down to ~6 nJ at the sample. The time-dependent PL signal arising after pump excitation was collected and focused onto a 0.5 mm thick β-BaB2O4 (BBO) crystal. Type-I sum frequency generation was achieved using a gate pulse of 800 nm. The tilt of the BBO crystal was adjusted to have maximum phase matching for the sum frequency process of the 800 nm gate pulse with the maximum of the PL signal at 545 nm. A motorized optical delay line on the gate line was used to scan the relative delay between the excitation and gate pulses. The upconverted signal was acquired with a thermally cooled (−80 °C) CCD detector (Halcyone, Sarasota, FL, USA) in a region of wavelengths corresponding to PL between 535 nm and 555 nm. 

**Time-resolved transmission measurements** were carried out using a time-resolved light transmission setup consisting of a Ti:Sapphire oscillator (Chameleon Ultra II, Coherent, Santa Clara, CA, USA) as the light source, producing a train of 140 fs pulses with a repetition rate of 80 MHz over the spectral range 680–1080 nm. The transmitted light through the sample was focused on the entrance slit of a spectrograph (Acton SP2300i, Princeton Instrument, Acton, MA, USA) coupled to a streak camera (Hamamatsu C5680, Hamatsu, Japan), resulting in a temporal resolution of about 3 ps.

## 4. Conclusions

In summary, this study presents a suitable and low-cost approach to fabricating colloidal photonic glasses based on a disordered assembly of polystyrene nanoparticles using the evaporation-assisted sedimentation method. SEM confirmed that monosized NPs are randomly arranged throughout the entire PG. Simultaneously, static transmittance measurements reveal a resonant behavior in diffuse light transport, associated with the presence of monosized particles that permit the collective excitation of the Mie resonance of the NPs. 

Subsequently, we spin-coated a DCM solution of the F8BT solution on top of the PG structure. We observed that the solution dissolves the PS nanoparticles in the first microns of the structure, allowing the polymer to reach the interface with the PG part left unchanged. Spectroscopic measurements, performed on F8BT polymer infiltrated in the PG, confirmed that the PG acts as a resonant photonic structure. In fact, the fluorescence of F8BT remains trapped inside for around 3 ps with respect to its counterpart based on a F8BT film, due to the diffusion constant of the structure. Moreover, the polymer infiltrated in the PG displays a narrowing of the full width at half maximum of the PL spectrum and an increase of almost 50% in its PL quantum yield. 

The results presented here suggest that this kind of PG structure can be used to enhance the spectroscopic features of the emitting polymer and could be considered a low-cost and suitable platform for the realization of visible lasers and fluorescence-based bio-sensors.

## Figures and Tables

**Table 1:** PL properties of F8BT/PG and F8BT film. Excitation wavelength: 405 nm. FWHM, full width half maximum; τ_av_, average lifetime obtained by best bi-exponential fits (see Figure S2); QY, PL quantum yield.

Label	PL Max (nm)	FWHM (cm^−1^)	τ_av_ (ns)	QY (%)
F8BT/PG	543	2545	1.46	37
F8BT film	543	2711	1.02	25

## Data Availability

The data presented in this study are available on request from the corresponding author.

## Supplementary Materials

The following supporting information can be downloaded at: https://www.mdpi.com/article/10.3390/molecules29030654/s1.
