# Supplementary material for: Enhancement of Photoluminescence Properties via Polymer Infiltration in a Colloidal Photonic Glass
Source: Molecules. 2024 Jan 30;29(3):654. doi: 10.3390/molecules29030654 (PMC10856319; doi:10.3390/molecules29030654)
Supplement: Supplementary file 1 [file molecules-29-00654-s001.zip › molecules-2820248-supplementary.pdf]

Supporting Materials

Characterization of the disorder by 2D FT analysis

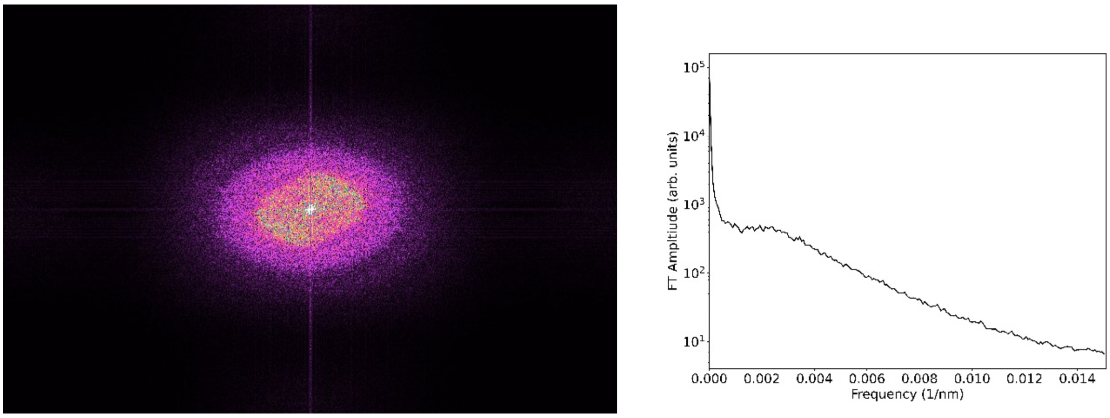

(a) **(b)**

Figure S1 a) 2D Fourier transforms of the corresponding colloidal assemblage; b) the power spectrum of the Fourier transform determined over radial average

Sample L1 image

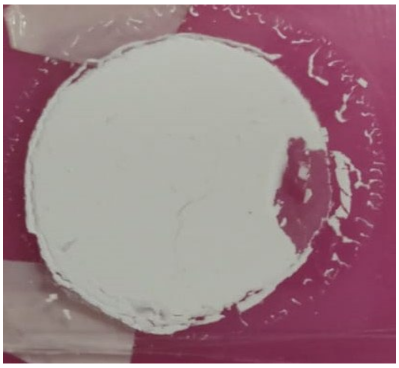

Figure S2: Image of the L1 sample.

Time-resolved Time-Correlated Single Photon Counting (TCSPC) measurements

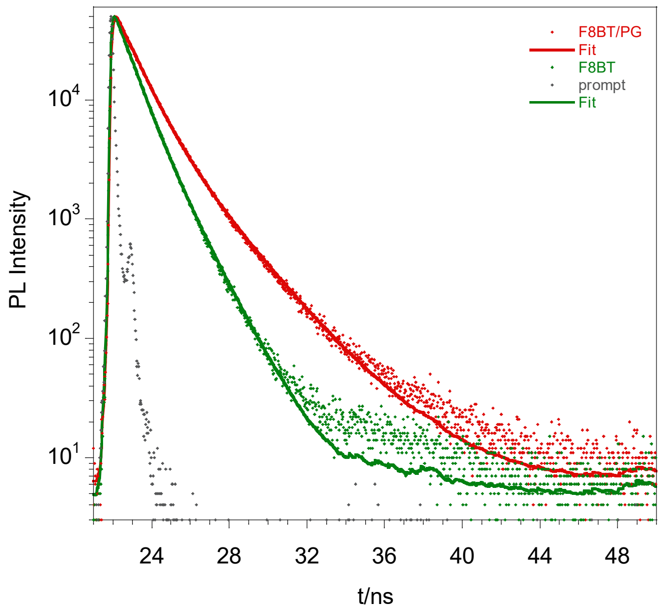

Figure S3 PL decays of F8BT/PG (red points) and F8BT film (green points), prompt (grey points) with biexponential fits (solid lines), excitation 405 nm. Parameters of the best fits are the following: F8BT/PG (0.82) 1.01ns; (0.18) 2.36ns, χ^2=2.21524. F8BT film (0.72) 0.75ns; (0.28) 1.41ns, χ^2=2.503447.

Streak camera measurements

Figure S4 shows the time resolved transmission of the 750 pump pulse through the PG structure. The temporal trace is compared with the instrument response showing no differences.

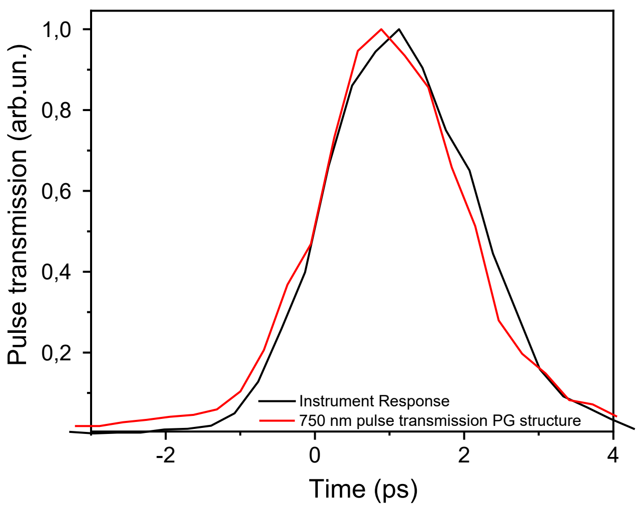

Figure S4: Time resolved transmission of the 750 nm pump pulse through the PG structure.
